# Pathological Complete Response After Neoadjuvant Chemotherapy in Breast Cancer: A Literature Overview

**DOI:** 10.3390/cancers18111718

**Published:** 2026-05-25

**Authors:** Anita Gorzelak-Magiera, Jacek Kabut, Joanna Sadurska, Anna Długaszek, Małgorzata Domagała-Haduch, Anna Szot, Iwona Gisterek-Grocholska

**Affiliations:** 1 Department of Oncology and Radiotherapy, Medical University of Silesia, 40-055 Katowice, Poland; 2 Students’ Scientific Association, Department of Oncology and Radiotherapy, Faculty of Medical Sciences in Katowice, Medical University of Silesia, 40-055 Katowice, Poland

**Keywords:** breast cancer, pathological response, residual disease, neoadjuvant treatment, neoadjuvant chemotherapy

## Abstract

Breast cancer is one of the leading causes of cancer deaths in women worldwide. Neoadjuvant chemotherapy (NACT) has increased rates of breast-conserving procedures and enabled the identification of patients with a particularly poor prognosis. Achieving a pathological complete response (pCR), an indicator of NACT efficacy, contrasts with residual disease (RD), which identifies patients at higher risk of recurrence. This review provides an overview of current evidence on the clinical and prognostic significance of pCR and RD in patients receiving NACT for breast cancer. The analysis is based on data from randomized clinical trials, meta-analyses, and current clinical guidelines for contemporary systemic treatment. Pathological complete response varies according to tumor subtype, with the highest rates observed in triple-negative and non-luminal HER2-positive breast cancer. In HER2-positive disease, the combination of chemotherapy with HER2-targeted therapies increases pCR rates, while the presence of RD supports escalation of postoperative treatment with antibody–drug conjugates. In triple-negative breast cancer (TNBC), the inclusion of platinum agents and immune checkpoint inhibitors improves treatment efficacy. In HER2-negative breast cancer and germline BRCA1/2 mutations, adjuvant PARP inhibitors improve survival independently of pCR, highlighting the complex relationship between pathological response and prognosis. Immunotherapy and targeted therapies are used alongside standard chemotherapy and hormone therapy in perioperative treatment. Further research is required to refine response assessment, integrate new biomarkers such as circulating tumor DNA (ctDNA), and optimize treatment selection, while clarifying the significance of reassessing hormone receptor and HER2 status in residual disease and its impact on subsequent treatment decisions.

## Simple Summary

Achieving a complete pathological response (pCR) after preoperative treatment for breast cancer is generally associated with a better prognosis, especially in HER2-positive and triple-negative subtypes. The proportion of patients obtaining complete pathological responses and the prognostic significance for patients are closely linked to the biological subtype of breast cancer. This paper presents the current state of knowledge regarding the significance of obtaining pCR following preoperative therapy. It discusses the role of anti-HER2 therapy in the perioperative management of the HER2-positive subtype, as well as the role of carboplatin and immunotherapy in triple-negative breast cancer. It also presents options for escalating adjuvant therapy following failure to achieve a complete pathological response in the postoperative report.

## 1. Background

Breast cancer is one of the leading causes of cancer-related morbidity and mortality [1]. An analysis by Joanne Kim et al. noted a 1–5% increase in the annual incidence of breast cancer in half of the countries studied, and also demonstrated a trend toward an increase in breast cancer incidence in younger age groups [2]. Risk factors for breast cancer include female gender, older age, pathogenic variants of breast cancer predisposition genes, family history of breast cancer, exposure to radiation involving the mammary glands, atypical hyperplasia and lobular carcinoma in situ, exogenous and endogenous estrogens, infertility, older age at first childbirth, alcohol consumption, obesity in postmenopausal women, physical inactivity, and short or no breastfeeding [1,3,4,5,6,7,8]. A positive trend toward a decrease in breast cancer mortality is noted in the most developed countries [2]. In recent years, numerous drugs have been introduced into clinical practice to improve overall survival in this group of patients. Currently, in addition to surgical treatment of early and locally advanced breast cancer, neoadjuvant chemotherapy (NACT), immunotherapy, HER-2 receptor-targeted therapy (anti-HER), CDK4/6 inhibitors, hormone therapy, and radiotherapy are used. This has become possible thanks to the understanding of predictive factors that allow the selection of patients who benefit from intensified treatment. Thus, breast cancer treatment has become an individualized therapy for a specific biological subtype of the tumor [9,10,11]. Determination of estrogen receptor (ER), progesterone receptor (PgR), and HER2 status is mandatory for all patients diagnosed with invasive breast cancer before initiating treatment. Treatment decisions are made based on the four main breast cancer subtypes: luminal A, luminal B HER2-positive, luminal B HER-2 negative, and triple-negative [9]. Table 1. The work by Tarantino et al. published in 2018 shed new light on the classic division of HHER2-positiveand HER-2 negative cancers, presenting the concept of HER-2 low tumors, which include tumors with HER-2 receptor overexpression of 1+ in immunohistochemistry (ISH) or 2+ with negative in situ hybridization (ISH) results. However, this is currently irrelevant in qualifying for preoperative treatment [9].

## 2. Methods

A literature review on the significance of achieving a complete pathological response following preoperative treatment was conducted between November 2025 and March 2026 using the PubMed, Scopus and Google Scholar databases. The following keywords and phrases were entered: “chemotherapy”, “residual disease”, “breast cancer” and “complete pathological response”. This enabled the creation of a preliminary list of potential sources, which were then subjected to a selection process based on titles and abstract content. The final selected studies were subjected to a thorough analysis and categorized according to the types of cancer they concerned. The types of articles selected included original research, literature reviews and meta-analyses. Most of the studies were written in English. The review provides information on preoperative therapy for breast cancer and its implications for long-term treatment outcomes, as well as methods for escalating therapy in the event of failure to achieve a complete pathological response. The review includes publications from 2005 to 2025. In turn, the new meta-analyses and review articles included in the text also take older studies into account.

A PICO framework was employed to define the eligibility criteria and guide data extraction and synthesis. The Population (P) consisted of adults with breast cancer, including both sexes where represented, across all histologic and molecular subtypes. The Intervention (I) encompassed any treatment regimens that led to the assessment of pCR, including neoadjuvant chemotherapy, targeted therapies, and immunotherapies. The Comparator (C) included diverse treatment regimens, standard versus experimental approaches, or studies without a direct comparator (as seen in observational work). The Outcome (O) focused on reported pCR, with explicit definitions used in each study (for example ypT0/is ypN0).

## 3. Results

Residual disease (RD) is typically defined as the presence of histopathologically confirmed cancer in the breast and/or lymph nodes after completion of neoadjuvant therapy. There is considerable variation in the definition of complete pathological response (pCR) across clinical trials. Some studies applied the definition of pCR only to the breast tumor, while others required a complete response in the axillary lymph nodes as well. Furthermore, some studies included the presence of focal invasive cancer or non-invasive residual cancer in their definition of pCR, while others defined the absence of all invasive and non-invasive cancer in the examined tissue sample. In recent years, pathological complete response (pCR) and lack thereof have become key endpoints in clinical trials and prognostic indicators for disease-free survival (DFS) and overall survival (OS) [10,11,12,13]. A meta-analysis of the Collaborative Trials in Neoadjuvant Breast Cancer (CTNeoBC) studies showed that patients with pCR have longer event-free survival (EFS) and overall survival. The association between complete pathological response after NACT and long-term outcomes is strongest in patients with triple-negative breast cancer and in patients with non-luminal HER2-positive tumors who received anti-HER therapy [14]. Recognition of this association has led to the recognition of pCR as an endpoint for accelerated approval of new drugs in neoadjuvant breast cancer by the US Food and Drug Administration (FDA) and the European Medicines Agency (EMA) [15]. In recent years, a number of clinical trials and meta-analyses have been published, which have deepened our understanding of the prognostic significance of pCR and the impact of RD on treatment decisions and outcomes. Predictive factors for achieving pCR, in addition to hormone receptor (HR) status and HER2 expression, include the tumor proliferative index.

### 3.1. Systems Defining Response to NACT

There are numerous scales for assessing response to NACT, and differences in the definition of complete pathological response (pCR) exist Table S1. This term can mean the absence of invasive cancer in the breast and lymph nodes according to the AJCC/UICC (TNM) classification, or the absence of invasive cancer in the breast and lymph nodes and the absence of carcinoma in situ according to the FDA German group [13]. The Miller-Payne system is a well-known system for assessing pathological response, but it only estimates the reduction in primary tumor cellularity after treatment, without taking into account tumor size and lymph node status. The reduction in cellularity is often greatest when the tumor is small, which is an important variable in assessing response to systemic therapy [16]. Symmans et al. assessed residual cancer burden (RCB), an indicator that takes into account the primary tumor size, its cellularity, and the percentage of carcinoma in situ in the primary tumor, as well as lymph node metastases, as a significant predictor of distant recurrence-free survival (DRFS). The estimated cumulative recurrence rate at 5 years was 5.4% for the pCR group (RCB-0) and 2.4% for the minimal RD group (RCB-I), while it was 53.6% for the extensive RD group (RCB-III), adjusted for competing deaths [17]. In the analysis by Wei Wang et al., both the RCB and Miller-Payne scores allowed for stratification of patient survival outcomes after NACT, but RCB demonstrated higher prediction accuracy, particularly in triple-negative breast cancer (TNBC) [18]. Despite the widespread use of the concepts of residual disease and complete pathological response, as well as the assessment of therapy efficacy in clinical trials based on residual tumor after preoperative therapy, this variable has its limitations. The analysis by Koen Kwakkenbos et al. aimed to compare ypTNM assessment practices based on a survey distributed to pathologists from different countries. The survey included general questions regarding the assessment of tumor diameter, as well as graphical scenarios presenting different tumor cell distributions. Data from 208 pathologists from 35 countries confirmed that although the ypTNM system is widely used, the reproducibility of results is limited, particularly in the assessment of the primary tumor (ypT). The lack of clear, standardized definitions is considered the main reason [19].

### 3.2. Neoadjuvant Chemotherapy (NACT)

In an article published in 1983, Fisher et al. demonstrated the potential superiority of chemotherapy before surgery in the form of improved tumor response and metastasis control in a mouse model, providing biological justification for this treatment sequence [20]. Initially, preoperative chemotherapy was used solely to reduce the advancement of the disease and facilitate surgical treatment or radical radiotherapy. Subsequent studies have demonstrated that in patients with both primarily resectable and inoperable tumors, the use of induction therapy is highly effective and increases the percentage of patients who can undergo less extensive surgery in both the breast and axillary region [21,22,23]. A meta-analysis of 14 randomized trials by Mieog et al. showed that patients receiving neoadjuvant chemotherapy had a significantly lower mastectomy rate compared to the group who underwent surgery first and then received adjuvant chemotherapy (relative risk 0.71 (95% confidence interval (CI) 0.67 to 0.75)) without affecting local control (hazard ratio 1.12 (95% CI 0.92 to 1.37)) [24]. The conducted analyses are limited by significant differences in the eligibility criteria, including the use of different treatment regimens and different clinical stages [25]. In 2008, the National Surgical Adjuvant Breast and Bowel Project (NSABP) published the results of Protocol B-18 and Protocol B-27 treatment, which were initiated in 1988. The 16-year follow-up of NSABP revealed no difference in overall survival (OS) or disease-free survival (DFS) in patients treated preoperatively with four cycles of AC (doxorubicin and cyclophosphamide) compared to those treated with postoperative chemotherapy. However, it is worth noting that a trend favoring preoperative chemotherapy for DFS and OS was observed in women under 50 years of age (P 0.09 for DFS; P 0.06 for OS). However, the addition of taxanes to preoperative treatment significantly increased the proportion of patients with pathological complete response (pCR) but did not affect DFS or OS. In both studies, patients who achieved pCR had significantly better DFS and OS outcomes compared with patients who did not achieve pCR [26]. Another year of improvement in preoperative chemotherapy outcomes was the use of dose-dense (DD) chemotherapy, which involves administering drugs at shorter intervals than in standard chemotherapy regimens. This is dictated by the biology and kinetics of cancer cell division. Increasing dose intensity, i.e., the amount of drug administered per unit of time, may contribute to increased cancer cell elimination, minimize the chance of tumor progression between chemotherapy cycles, and thus reduce the risk of treatment failure [27]. Importantly, the risk of adverse events during dose-dense chemotherapy was comparable to the risk of adverse events during conventional fractionated chemotherapy. A large meta-analysis published in 2019 comparing dose-dense regimens showed fewer breast cancer recurrences in the higher-dose-density group than in the standard chemotherapy regimen group, and a reduction in breast cancer mortality, as well as all-cause mortality [28]. A biweekly dose-dense regimen of doxorubicin and cyclophosphamide combined with granulocyte colony-stimulating factor (G-CSF) was originally an option for the adjuvant treatment of HER2-negative breast cancer with a high proliferation index [29,30]. Despite the lack of solid evidence supporting the role of dose-dense chemotherapy in the neoadjuvant setting, the European Society of Clinical Oncology and the National Comprehensive Cancer Network have extended this recommendation to neoadjuvant treatment [31,32]. Although small differences in the efficacy of preoperative and postoperative chemotherapy are noted, NACT has become well-established in clinical practice. The advantages of neoadjuvant treatment include its potential impact on the early treatment of micrometastases, allowing time for genetic testing, and enabling the assessment of tumor response to chemotherapy, which correlates with its chemosensitivity and long-term treatment outcomes [33,34]. Postoperative assessment allows for a decision to escalate systemic treatment based on the response. The impact of preoperative chemotherapy on downstaging of axillary cancer is also becoming an increasingly important factor in deciding on this approach, as resection can be avoided in some patients who had N1 disease prior to induction therapy.

### 3.3. Perioperative Treatment of Patients with HER2-Positive Breast Cancer

Residual disease in HER2-positive breast cancer is classified according to the Residual Cancer Burden (RCB) scale. Patients with pCR (RCB-0) demonstrate significantly better long-term outcomes compared to patients with residual disease (RCB-I, RCB-II, RCB-III). The highest pCR rates are achieved in HR-negative, HER2-positive tumors when trastuzumab is added to multi-agent chemotherapy regimens [11]. The NOAH study was the first to demonstrate that the addition of trastuzumab to neoadjuvant chemotherapy and continued adjuvant improves EFS and pCR. In the follow-up assessment, a significant trend in the final OS study was observed: 5-year OS was 73.5% vs. 62.9%, and the conclusion for the OS result was *p* = 0.055 [35]. Another significant study was GeparQuattro, in which patients received four cycles of epirubicin/cyclophosphamide, followed by four cycles of docetaxel with or without capecitabine and trastuzumab every 3 weeks during all chemotherapy cycles. Of the 1509 study participants, 445 had HER2-positive tumors. The addition of trastuzumab was investigated as a second primary end point by an indirect comparison of patients with HER2-positive disease when treated in addition to trastuzumab with a reference group of patients with HER2-negative tumors treated with chemotherapy alone. The pathological complete response rate, defined in the protocol as the absence of invasive or in situ residual tumor lesions, was 31.7%, which was 16% higher than in the control group [36]. The addition of trastuzumab to neoadjuvant chemotherapy and the postsurgical phase lead to a similar DFS of patients with HER2-positive disease when compared with a reference group of patients with HER2-negative disease receiving chemotherapy alone [37]. A significant limitation of the study was the design of the comparison groups. Patients with HER2-positive tumors treated with neoadjuvant trastuzumab were compared with a group of HER2-negative patients, not with a HER2-positive cohort not receiving anti-HER2 therapy. This study design limits the ability to clearly attribute the observed therapeutic differences solely to the effect of trastuzumab. However, these studies did not incorporate dual anti-HER2 blockade, which is currently regarded as the most effective therapeutic approach in HER2-positive breast cancer [37].

In the Neosphere 2 phase study, which enrolled 417 patients with HER2-positive breast cancer, stage T2-3, N0-3, without distant metastases, the highest complete pathological response rate was observed in the arm with dual anti-HER2 blockade (pertuzumab, trastuzumab) combined with docetaxel (45.8% vs. 29%). Importantly, the pCR rate was higher with docetaxel, trastuzumab, and pertuzumab in patients with ER−/HER2+ cancer (63.2%) compared to patients with ER+/HER2+ cancer (26%) [38]. The primary objective of the TRYPHANEA study was to evaluate safety and tolerability during neoadjuvant treatment but it also demonstrated a high pCR rate when dual HER2 receptor blockade was added to neoadjuvant chemotherapy. In the study, 225 patients with resectable, locally advanced, or inflammatory breast cancer were randomly assigned to one of three treatment arms: Arm A: 5-fluorouracil, epirubicin, cyclophosphamide (FEC) followed by docetaxel (T), with trastuzumab (H) and pertuzumab (P) given concurrently throughout (FEC + H + P × 3 → T + H + P × 3); Arm B: FEC followed by T + H + P (FEC × 3 → T + H + P × 3); or Arm C: T, carboplatin, H with P (TCH + P × 6). The highest pCR rate (ypT0/is) was reported in the TCH + P arm, at 66.2% compared with 61.6% (Arm A) and 57.3% (Arm B). The pCR rate was higher in patients with hormone receptor-negative tumors compared with patients with hormone receptor-positive tumors [39].

Adding lapatinib to trastuzumab and paclitaxel was another approach to improving outcomes in the neoadjuvant setting. In the randomized phase 3 NeoALTTO trial, 445 patients with HER2-positive breast cancer were randomly assigned to receive lapatinib or trastuzumab, or the combination. Anti-HER2 therapy was administered alone for the first 6 weeks; then, weekly paclitaxel was added to the regimen for the next 12 weeks, after which patients underwent surgery, followed by continued adjuvant chemotherapy and the same targeted therapy as in the neoadjuvant setting. The pCR rate was significantly higher in the lapatinib and trastuzumab group than in the group given trastuzumab alone (51.3% vs. 29.5%). Efficacy did not differ significantly between the lapatinib and trastuzumab groups. Landmark analyses showed that 3-year event-free survival was significantly improved for women who achieved pathological complete response compared with those who did not, as was 3-year overall survival [40].

Neoadjuvant chemotherapy with HER-2 receptor-targeted therapy in patients with ER+/HER2+ tumors is less likely to achieve pathological complete response (pCR) than in non-luminal HER2-positive patients. This formed the basis for the design of the Paltan study, which recruited patients with ER+/HER2+ breast cancer. The study aimed to synergistically inhibit both hormone-dependent receptors and the HER-2 receptor. It combined palbociclib, letrozole, and trastuzumab (PLT) as neoadjuvant therapy in early breast cancer. The primary endpoint was pCR assessed at 16 weeks. However, the study was stopped prematurely due to lack of efficacy. The pCR rate achieved was only 7.7% [41]. Another study using a chemotherapy-free approach was NA-PHER2. Six cycles of dual HER2 blockade using trastuzumab and pertuzumab and five cycles of palbociclib and fulvestrant were used in this study. The study achieved a pCR rate of 27% [42]. Similar disappointing results were achieved in the TBCRC 006 study, where the pCR rate with dual HER2 blockade using trastuzumab, lapatinib, and hormonal therapy was only 21% in patients with ER+/HER2+ cancer and 36% in patients with ER-/HER2+ cancer [43] (Table 2).

The PHERGain study aimed to identify patients with HER2-positive, invasive, operable stage I-IIIA breast cancer with at least one lesion evaluable on PET scan, who could avoid chemotherapy. Patients were randomly assigned to receive docetaxel, carboplatin, trastuzumab, and pertuzumab (TCHP) or to group B, where patients received trastuzumab and pertuzumab with or without hormonal therapy, and the decision to administer chemotherapy was based on the PET scan results. Responding patients continued treatment with trastuzumab and pertuzumab, while non-responding patients received six cycles of TCHP chemotherapy. Ninety-four percent of patients who adopted this adaptive treatment strategy, regardless of whether they received chemotherapy or not, experienced no recurrence of iDFS three years after surgery [44].

Tumor-infiltrating lymphocytes (TILs) have predictive value for response to neoadjuvant chemotherapy in triple-negative breast cancer (TNBC) and HER2-positive breast cancer. In the analysis of patients with HER2-positive breast cancer, the pCR rates were 32%, 39%, and 48% in the groups of patients with low, intermediate, and high TIL levels, respectively [45].

A study by Yibin Qiu published in 2026 demonstrated that the level of HER2 receptor expression in patients treated preoperatively and previously considered HER2-negative is not neutral. The pCR rates in the study were 8.4% for HER2-low, 20.4% for HER2-ultralow (HER2 1+), and 25.0% for HER2-null, respectively. HER2 expression correlated inversely with pCR rates. At a 70.8-month follow-up, survival analysis showed that higher HER2 expression was associated with significantly better iDFS and OS. Therefore, these findings suggest that HER2 expression level (low/ultralow/null) stratifies prognosis in HER2-negative early breast cancer [46]. Nevertheless, further prospective studies are required to validate these observations and to clarify their clinical significance.

The identification of residual disease as a negative prognostic factor prompted researchers to attempt escalation of adjuvant treatment. The breakthrough phase III KATHERINE study demonstrated that replacing postoperative trastuzumab with the antibody-drug conjugate ado-trastuzumab emtansine (T-DM1) in patients who failed to achieve pCR after neoadjuvant chemotherapy with anti-HER2 therapy (single or dual HER2 blockade) resulted in an 11% absolute improvement in iDFS (3-year iDFS 88% vs. 77%). Subgroup analyses confirmed the benefit, regardless of hormone receptor status, disease burden at the time of surgery, and prior HER2-targeted neoadjuvant therapy [47].

In October 2025, the results of the DESTINY-Breast 05 study were published.

This study compared treatment with trastuzumab deruxtecan with trastuzumab emtansine in patients with residual invasive disease or lymph node metastases at the time of surgery. The study enrolled 1635 patients. The 3-year disease-free survival was 92.3% and 83.5%, respectively. A significant issue was the incidence of confirmed interstitial lung disease related to T-DXd treatment (9.6% vs. 1.6%), which resulted in death in two patients [48] (Table 3). 

### 3.4. Perioperative Treatment of Triple-Negative Breast Cancer

TNBC accounts for 10–15% of breast cancers. Biologically, these tumors often exhibit high histological grade G3 and a high Ki67 proliferation index. They are more often diagnosed in more advanced stages and in younger patients. Up to 20–25% of patients with this biological subtype have BRCA mutations (more often BRCA1) [57]. Compared to other subtypes, patients with TNBC have significantly higher pCR rates after NACT but lower 3-year progression-free survival and 3-year overall survival (OS). If pCR is achieved in this group of patients, TNBC patients have similar survival times to patients with other biological subtypes, whereas patients with this biological subtype who do not achieve pCR have shorter overall survival times (P 0.0001) [58]. In neoadjuvant treatment of triple-negative breast cancer, the addition of carboplatin has been shown to positively impact complete response rates. In the GepardSixto study, the addition of carboplatin significantly improved the pCR rate (53% vs. 37%). Patients were treated for 18 weeks with paclitaxel and non-pegylated liposomal doxorubicin and TNBC received simultaneous bevacizumab [59]. Although GeparSixto trial was criticized for adopting a non-standard regimen, its results were further validated in the CALGB 40603 trial, which enrolled 443 TNBC patients and adopted a standard neoadjuvant regimen with weekly paclitaxel followed by dose-dense doxorubicin plus cyclophosphamide with the addition of carboplatin and/or bevacizumab. Carboplatin group reached pCR rate up to 54% compared to control group with 41%. Both trials shed light on the clinical application of platinum for TNBC [60]. Another attempt to improve treatment outcomes was the BrighTNess study, in which carboplatin, with or without veliparib (a polyADP-ribose polymerase inhibitor), was added to chemotherapy based on anthracycline, cyclophosphamide, and then paclitaxel. The addition of carboplatin significantly improved the pCR rate, unlike veliparib, which had no impact on treatment efficacy. Furthermore, this study demonstrated that the addition of carboplatin is particularly important for patients without BRCA 1 or BRCA 2 mutations [49]. Gupta et al. [50] demonstrated the effect of carboplatin on improving EFS and OS in younger women. In a population of 717 patients, the addition of a weekly dose of carboplatin to chemotherapy based on anthracycline, cyclophosphamide, and paclitaxel resulted in an absolute increase in 5-year relapse-free survival. Carboplatin resulted in a statistically significant increase in complete pathological response in the breast and lymph nodes. This result was achieved by 40.3% of patients in the control group and 54.5% in the carboplatin group, with the benefit primarily occurring in younger patients (41.5% vs. 61.0%, *p* < 0.001) [50].

Another attempt to increase the pCR rate, this time using immunotherapy, was the Keynote-522 study. The study included 1174 patients with previously untreated stage II or III TNBC, randomized to preoperative treatment with paclitaxel and carboplatin, followed by four cycles of doxorubicin or epirubicin and cyclophosphamide. In the experimental arm, pembrolizumab was additionally used both preoperatively (8 doses every 3 weeks) and postoperatively (9 consecutive doses). In the primary analysis, which included 602 patients, the pCR rate was 64.8% in the pembrolizumab group compared to 51.2% in the placebo group (*p* < 0.001). The 3-year EFS rate was 84.5% vs. 76.8% in the placebo group (*p* < 0.001) (Table 4). However, adjuvant capecitabine was not incorporated into the trial design. Although the Capecitabine for Residual Cancer as Adjuvant Therapy (CREATE-X) trial showed that adjuvant capecitabine prolonged survival among patients with triple-negative breast cancer, the present trial was designed before these results were reported [51,53].

Reassessment of pCR in the entire study population indicated an advantage for immunotherapy, although the numerical difference was smaller (7.4%). Compared to placebo, pembrolizumab moved patients to lower RCB categories.

In the pembrolizumab group, there were more patients with RCB-0 (pCR) and fewer with RCB-1, RCB-2, and RCB-3. Furthermore, the addition of pembrolizumab to chemotherapy resulted in fewer events (EFS) in the RCB-0, RCB-1, and RCB-2 categories. The greatest benefit was observed in the RCB-2 category [51]. Due to the fact that the Keynote-522 trial used traditional AC dose (administered every 3 weeks), an attempt was made to further improve treatment outcomes by determining the value of dose-dense doxorubicin and cyclophosphamide. A meta-analysis published in 2026 analyzed four observational studies, including a total of 535 patients diagnosed with early triple-negative breast cancer. No significant differences in pCR were observed between AC administered every 2 weeks and AC administered every 3 weeks. More serious adverse events were reported among patients receiving ddAC [61].

The results of the GeparDouze trial, presented at SABCS2024, did not demonstrate a significant improvement in event-free survival (EFS) when atezolizumab was added to neoadjuvant chemotherapy followed by adjuvant immunotherapy. The exception was the group of node-positive patients, who appeared to benefit. Despite a higher pCR rate in the immunotherapy arm, no OS benefit was observed after 4 years of atezolizumab treatment [52].

However, a systematic Cochrane review that included two trials combining PD-1 inhibitors and five trials combining PD-L1 inhibitors, with a total of 4341 participants, demonstrated that combining checkpoint inhibitors with chemotherapy before breast cancer surgery improves pathological response, event-free survival (EFS), and overall survival (OS) in early triple-negative breast cancer (TNBC), while having marginal significance in the adjuvant setting [62].

As in HER-2-positive tumors, the level of TIL infiltration correlates with the chance of achieving a complete pathological response. This is 31% for patients with low TILs, 31% for those with intermediate TILs, and 50% for those with high TILs, respectively [45].

Despite high chemosensitivity and a significant pCR rate after neoadjuvant chemotherapy, the risk of relapse in the first years after diagnosis is high. Triple-negative tumors have a higher incidence of distant metastases to visceral organs and to the brain, in contrast to hormone receptor-positive tumors, which have a higher tendency to metastasize to bone [63]. The first study to change the standard of care for patients with residual disease was the Create-X trial. The pathological effect of neoadjuvant chemotherapy was graded on a scale of 0 to 3 according to the Japanese Breast Cancer Society response criteria. In patients with both luminal and triple-negative breast cancers who had received standard neoadjuvant chemotherapy containing an anthracycline, a taxane, or both, the addition of 6 to 8 cycles of capecitabine chemotherapy was found to be safe and effective in prolonging disease-free survival (74.1% vs. 67.6% of the patients were alive and free from recurrence or second cancer at 5 years) and overall survival (89.2% vs. 83.6% of the patients were alive at 5 years). Among patients with triple-negative disease, DFS was 69.8% vs. 56.1% in the control group, and OS was 78.8% vs. 70.3%, indicating comparatively better outcomes in the TNBC subgroup than in the overall study population [53].

Based on an analysis published in 2025, it appears that the efficacy of treating patients with TNBC and residual disease may be improved by adding bevacizumab to capecitabine. In this analysis, the highest 36-month RFS rate was achieved in the bevacizumab plus capecitabine arm at a dose based on body surface area (BSA) (75.0%) [64].

The SYSUCC-001 study attempted to test the efficacy of capecitabine maintenance therapy administered for one year. The study met its primary endpoint of DFS, with a 5-year rate of 82.8% in the capecitabine group vs. 73% in the control group. Superiority was also observed in DDFS (85.8% vs. 75.8%) and OS (85.5% vs. 81.3%) [65]. Not all studies using capecitabine in the adjuvant setting for residual disease have been successful. An example is the GEICAM/2003-10 study, which failed to achieve the targeted improvement in DFS [54].

The BREASTIMMUNE-03 study attempted to utilize immunotherapy in patients with residual disease. It compared the combination of nivolumab with ipilimumab versus capecitabine. The study was negative. Dual immunotherapy did not demonstrate a DFS advantage over capecitabine as adjuvant treatment in patients with RCB II-III TNBC after NAC that did not include ICIs [66]. A prospective Phase III study is currently underway, the SASCIA study, in HER2-negative breast cancer with residual disease after NACT. Patients were randomly assigned to receive sacituzumab or investigator-selected treatment. Patients with HR-positive breast cancer were scheduled for hormonal therapy, in accordance with local guidelines. The results aim to answer the question of whether the use of the conjugate will improve prognosis in patients who do not achieve pCR [67].

The Phase III AFT-65/ASCENT-05/OptimICE-RD study aims to evaluate the efficacy and safety of sacituzumab govitecan in combination with pembrolizumab compared with physician’s choice of treatment (pembrolizumab ± capecitabine) in patients with early-stage TNBC with residual invasive disease after neoadjuvant therapy [68].

### 3.5. Treatment of Residual Disease in Patients with BRCA Mutations

Olaparib is the first oral, first-generation inhibitor of poly-ADP-ribose polymerase (PARP 1 and 2) enzymes, which play a key role in the repair of single-stranded DNA breaks by base excision. In a normal cell, blocking the PARP1 repair protein has no effect because it switches the DNA repair mechanism to homologous recombination. Mutations in the genes encoding BRCA 1 and 2 proteins, which are key to homologous recombination (HRD), result in a loss of repair capacity and the accumulation of damage, transforming into double-stranded DNA breaks, which the cancer cell cannot effectively repair, leading to apoptosis [69]. iPARPs are the first targeted drugs in oncology to utilize the concept of synthetic lethality, which involves the loss of a gene or protein in a cancer cell. Cancer cells with mutations in BRCA 1 or 2 genes are dependent on DNA repair mechanisms other than homologous recombination, so blocking PARP proteins is a mechanism leading to their death [70]. The first study that led to olaparib’s registration by the European Medicines Agency (EMA) was published in the New England Journal of Medicine (NEJM) in 2012. This study involved a large group of patients with relapsed, platinum-sensitive, poorly differentiated serous ovarian cancer who responded to rechallenge with platinum-based chemotherapy [71]. After completion of the study, patients were randomly assigned to receive olaparib or placebo as maintenance therapy. The results were positive regardless of BRCA mutation status. Subgroup analysis revealed that olaparib was even more beneficial in patients with a BRCA mutation (germline or somatic).

The basis for the registration of olaparib in the adjuvant treatment of breast cancer was the OlympiA study—a randomized, double-blind, phase III study that compared one year of adjuvant treatment with olaparib with placebo in 1836 patients with a pathogenic/likely pathogenic germline mutation in the BRCA1/2 genes, early HER2-negative breast cancer, and high risk of recurrence [55]. All patients underwent standard chemotherapy (at least 6 cycles preoperatively or postoperatively), surgery, and radiotherapy—local treatment had to be completed no earlier than 2 and no later than 12 weeks before study entry. For patients who received preoperative treatment, the eligibility criterion for the study was the absence of a pathological complete response (pCR) in the postoperative material. For hormone-dependent, HER2-negative patients treated with adjuvant chemotherapy, the criterion for inclusion in olaparib treatment was at least four positive lymph nodes, whereas for the group with triple-negative breast cancer treated adjuvantly, N+ status or a tumor at least 2 cm in size was a prerequisite for inclusion in the olaparib arm. The first interim analysis demonstrated a significant reduction in IDFS and DDFS in the olaparib arm compared with placebo, translating into an increase in 3-year IDFS and DDFS by approximately 8–9 percentage points. An updated second OS analysis (median follow-up 3.5 years) demonstrated a significant improvement in overall survival (HR 0.68; *p* = 0.009), with a 4-year OS of 89.8% vs. 86.4% favoring olaparib and sustained improvements in IDFS and DDFS with no new safety events. Treatment benefit was observed in most subgroup analyses, regardless of subtype (TNBC vs. HR-positive). Quality of life analysis showed that olaparib was generally well tolerated, with no significant long-term deterioration in quality of life compared with placebo. In practice, pCR in the postoperative study is a risk stratification criterion: a patient with pCR usually has a prognosis sufficiently good to be ineligible for olaparib, while lack of pCR (high residual disease) is one of the key indications for initiating therapy. In contrast to the OlympiA study, in 2025, the phase II/III PARTNER study was published in Nature, which directly assessed the effect of adding olaparib to standard neoadjuvant chemotherapy based on anthracyclines, taxanes, and carboplatin on pCR rate and survival in patients with breast cancer with a BRCA1 or 2 mutation. The results showed that although the addition of olaparib did not increase pCR in the group of patients with a BRCA mutation, it improved event-free survival (EFS) and OS in this group and revealed a lack of a close association between pCR and survival in the BRCA 1/2 genes mutations subtype [56]. This may suggest a complex effect of various molecular and predictive factors.

### 3.6. Future Research Directions

A challenge remains that residual disease may differ from pre-treatment tumors, and NAC may not completely eliminate the cancer, but instead select for resistant clones and promote the development of transcriptomic and molecular changes [72]. Among the predictors of achieving complete pathological response, in addition to biological subtype and estrogen receptor status, changes in the Ki67 proliferation index and the level of tumor-infiltrating lymphocytes (TILs) are also mentioned [73,74]. A new approach to selecting patients at particularly high risk of relapse is monitoring free circulating DNA. In the study by Natasha B. Hunter et al., ctDNA detection after NAT was prognostic for recurrence, regardless of achieving pCR after neoadjuvant treatment. Furthermore, ctDNA detection after the procedure enabled the identification of patients at exceptionally high risk of disease recurrence [75].

## 4. Discussion

Although the prognosis for breast cancer patients is steadily improving, the high mortality rate from this cancer raises the need to explore new methods to improve the prognosis for this group of patients.

Preoperative chemotherapy is the standard of care for most patients with HER2-positive and triple-negative breast cancer, except for very early-stage breast cancers [31].

According to NCCN guidelines and the CTNeoBC pooled analysis, the correlation between pathologic response and long-term outcomes is strongest for TNBC, weaker for HER2+, and weakest for ER-positive disease [14,76,77]. The GepardQuatro study, which analyzed the addition of capecitabine and trastuzumab to EC-T chemotherapy, provided controversy. As a secondary primary endpoint, patients with HER2-positive disease treated with trastuzumab were indirectly compared to a reference group of patients with HER2-negative tumors treated with chemotherapy alone. Eligibility for treatment encompassed a wide range of patients and was influenced by subjective assessment. Patients treated with trastuzumab with HER2-positive disease demonstrated similar DFS but significantly better adjusted OS compared to patients with HER2-negative disease treated with chemotherapy alone [36]. This demonstrates that extrapolating benefits from primary endpoints does not always translate into overall survival benefit or loss. Biological heterogeneity within HER2-positive disease is a well-established phenomenon with profound implications for treatment response and the interpretation of pCR. ER−/HER-2+ and ER+/HER2+ tumors represent biologically distinct entities rather than a single disease, as evidenced by consistently divergent pCR rates across multiple landmark trials and large-scale analyses. In the EA1181 trial (*n* = 2141), pCR rates were 63.7% for HER2+/ER− versus 32.4% for HER2+/ER+ tumors, with an inverse relationship between ER expression level and pCR likelihood (ER 1–10%: 62.5%; ER 11–69%: 51.6%; ER ≥ 70%: 22.5%) [78]. Critically, the prognostic significance of pCR itself differs by ER status: a pooled analysis of CALGB 40601, NeoALTTO, and NSABP B-41 demonstrated that pCR was significantly associated with event-free survival only in HER2-enriched and basal-like subtypes, but not in luminal and/or ER-positive tumors, where luminal gene expression signatures predicted favorable long-term outcomes, even in the absence of pCR [79].

The results of the KATHERINE study were a breakthrough for patients with HER-2-positive breast cancer and residua disease, which were the basis for the registration of trastuzumab emtansine for this indication [47]. However, in a study comparing trastuzumab emtansine with trastuzumab deruxtecan, it was found that switching the conjugate may lead to further improvement in treatment outcomes in this group of patients [48]. These outcomes are expected to shortly constitute the standard of care for patients in this cohort.

The Keynote 522 trial demonstrated the value of immunotherapy in preoperative treatment, which has changed the standard of care. The absolute difference in the 36-month EFS rate between patients receiving pembrolizumab versus placebo after achieving CR0 was 1.9%, while the prognosis remained poor in patients with RCB 2, and especially with RCB 3. The study did not allow the use of capecitabine following neoadjuvant therapy. This raises doubts as to whether the use of ICIs in adjuvant therapy provides measurable benefits.

The investigators chose complete pathological response rate as their primary endpoint, which does not always correlate with overall survival. This may be particularly relevant in the context of immunotherapy, where treatment effects are partially associated with modulation of the tumor microenvironment and effects on micrometastatic disease [51]. An example is the GeparDouze study, in which atezolizumab was added to neoadjuvant chemotherapy, which increased the pathological complete response (pCR) rate from 57% to 63% compared to placebo, but did not translate into improved 4- EFS rate (85.2% vs. 81.9%) or at 4 years OS (90.2% vs. 89.5%) [52]. Biologic differences between anti-programmed cell death 1 and anti-programmed death ligand 1 (PD-L1) antibodies might partially explain the differences in outcomes between the GeparDouze study and Keynote-522 investigating the benefit of neoadjuvant immunotherapy plus chemotherapy in patients with TNBC.

The CREATE-X trial provided the first evidence supporting the efficacy of adjuvant therapy in patients with the RCB. It demonstrated an improvement in DFS and OS, which translated into a significant benefit exclusively in patients with TNBC. Literature reviews cited in the ESMO guidelines showed that adjuvant capecitabine improved OS, reducing the relative risk by 12–30% in this patient group, but there is limited evidence of its efficacy in patients with HR-positive disease. There is no data on whether capecitabine following neoadjuvant therapy provides additional benefits to patients who continue treatment with an ICI or olaparib after neoadjuvant therapy. The safety profile of the combination of these drugs has also not been tested. The study did not analyze the impact of BRCA mutations on the efficacy of adjuvant capecitabine therapy. Notably, the clinical response to neoadjuvant chemotherapy was evaluated using the Japanese Breast Cancer Society’s rarely used response criteria, and patients received neoadjuvant chemotherapy with a range of regimens [31,53].

The OlympiA study focused on patients with germline BRCA mutations (gBRCA) and high-risk breast cancer. Olaparib significantly improved overall survival, regardless of tumor subtype, chemotherapy regimen, or BRCA mutation type (BRCA1 or BRCA2). In patients with TNBC, olaparib was associated with a 4-year absolute improvement in overall survival of 3.8%. As in previous studies, the protocol did not allow the use of pembrolizumab and capecitabine [55]. The CREATE-X, KEYNOTE-522, and OlympiA trials all demonstrated positive results with a treatment escalation strategy after neoadjuvant chemotherapy in selected patients with TNBC and residual disease after neoadjuvant chemotherapy. Unfortunately, all three studies emphasize the need for improved treatment strategies, as patients with poor response to neoadjuvant therapy continue to have unfavorable prognoses. The current study design does not permit both sequential and concurrent administration of immunotherapy, chemotherapy, and PARP inhibitors, thereby requiring extrapolation of safety and efficacy from metastatic-population studies [51,53,55].

In luminal (HR+/HER2−) early breast cancer, the role of pCR is limited. Luminal tumors achieve pCR after neoadjuvant chemotherapy far less frequently than other subtypes. In the EORTC 10994/BIG 1-00 trial pCR was achieved in only 7.5% of luminal A and 15% of luminal B/HER2− tumors. Lower ER expression, higher histological grade, higher Ki-67, and luminal B subtype increase the possibility of achieving pCR, but are also associated with worse overall prognosis [79].

A significant limitation of pCR is the lack of standardized definitions and pathological assessment methods, which limits direct comparison of results across studies [14,80,81]. The CTNeoBC analysis demonstrated that complete eradication of invasive disease in both the breast and lymph nodes was more strongly associated with improved EFS and OS than eradication of disease in the breast alone [14]. However, long-term outcomes are largely determined by undetectable micrometastatic disease, meaning that eradication of the primary tumor does not necessarily reflect the overall efficacy of treatment [80].

## 5. Conclusions

The multitude of studies on breast cancer allows for an increasingly better understanding of the factors that influence the assessment of treatment efficacy. However, further research is needed to translate this knowledge into clinical practice and optimize the selection of adjuvant therapy to prolong survival in this group of patients.

## Figures and Tables

**Table 1:** Basic division of biological subtypes of breast cancer [9].

Breast Cancer Subtype	Luminal Breast Cancer				Non-Luminal Breast Cancer	
	Luminal A	Luminal B HER2-Positive	Luminal B HER2-Negative		Non-Luminal HER2-Positive	Triple-Negative Breast Cancer
Estrogen receptors (ER)	present	present	present		none	none
Progesteron receptors (PgR)	≥20%	each	<20%	each	none	none
HER2	none	present	none	none	present	none
Ki-67	<20%	each	each	≥20%	each	each

**Table 2:** Characteristics and outcomes of selected neoadjuvant clinical trials in HER2-positive early breast cancer.

Study	HER2 Status	Treatment	*n*	pCR (%)
NOAH [35]	HER2+	Doxorubicin + paclitaxel + cyclophosphamide + methotrexate + fluorouracil + trastuzumab	117	38
	HER2+	Doxorubicin + paclitaxel + cyclophosphamide + methotrexate + fluorouracil	118	19
	HER2−	Doxorubicin + paclitaxel + cyclophosphamide + methotrexate + fluorouracil	99	16
GeparQuattro [36]	HER2+	Epirubicin + cyclophosphamide → docetaxel ± capecitabine + trastuzumab	445	31.7
	HER2−	Epirubicin + cyclophosphamide → docetaxel ± capecitabine	1050	15.7
NeoSphere [38]	HER2+	Trastuzumab + docetaksel	107	29
	HER2+	Pertuzumab, trastuzumab + docetaksel	107	45.8
	HER2+	Pertuzumab +trastuzumab	107	16.8
	HER2+	Pertuzumab + docetaksel	96	24
TRYPHAENA [39]	HER2+	5-fluorouracyl + epirubicin + cyclophosphamide + trastuzumab + pertuzumab → docetaxel + trastuzumab + pertuzumab	73	61.6
	HER2+	5-fluorouracyl + epirubicin + cyclophosphamide → docetaxel + trastuzumab + pertuzumab	75	57.3
	HER2+	Docetaxel + carboplatin + trastuzumab + pertuzumab	77	66.2
NeoALTTO [40]	HER2+	Lapatinib → paclitaxel	154	24.7
	HER2+	Trastuzumab → paclitaxel	149	29.5
	HER2+	Lapatinib + trastuzumab → paclitaxel	152	51.3
PALTAN [41]	HER2+	Palbociclib + letrozole + trastuzumab	26	7.7
NA-PHER2 [42]	HER2+	Palbociclib + fulvestrant + trastuzumab + pertuzumab	30	27
TBCRC 006 [43]	HER2+	Trastuzumab + lapatinib ± letrozol	64	27
PHERGain [44]	HER2+	Trastuzumab + pertuzumab ± endocrine therapy (letrozole or tamoxifen)	227	38

**Table 3:** Summary of studies on NACT and residual disease.

Study	Phase	Treatment	*n*	Survival Endpoint	Survival Outcome	HR	*p*-Value
KATHERINE [47]	III	Trastuzumab + emtansine	743	3-year iDFS	88.3%	0.50; 95% CI 0.39–0.64	*p* < 0.001
		Trastuzumab	743		77%		
DESTINY-Breast 05 [48]	III	Trastuzumab + deruxtecan	818	3-year iDFS	92.4%	0.47; 95% CI 0.34–0.66	*p* < 0.001
		Trastuzumab + emtansine	817		83.7%		
BrighTNess [49]	III	Paclitaxel	158	4-year EFS	69%	-	
		Paclitaxel + carboplatin	160		79%	0.57; 95% CI 0.36–0.91 vs. paclitaxel	*p* = 0.02
		Paclitaxel + carboplatin + veliparib	316		78%	0.63; 95% CI 0.43–0.92 vs. paclitaxel	*p* = 0.02
Gupta et al., 2026 [50]	III	Paclitaxel + carboplatin → doxorubicin or epirubicin + cyclophosphamide vs. Paclitaxel → doxorubicin or epirubicin + cyclophosphamide	361 vs. 356	5-year EFS	70.7% vs. 64.1%	0.80; 95% CI 0.62–1.03	*p* = 0.081
				5-year OS	74.4% vs. 66.8%	0.74; 95% CI 0.57–0.97	*p* = 0.029
KEYNOTE-522 [51]	III	Pembrolizumab + paclitaxel + carboplatin → pembrolizumab + doxorubicin/epirubicin + cyclophosphamide Adjuvant pembrolizumab vs. Paclitaxel + carboplatin → doxorubicin/epirubicin + cyclophosphamide	784 vs. 390	5-year EFS	81.2% vs. 72.2%	0.65; 95% CI 0.51–0.83	-
				5-year OS	86.6% vs. 81.7%	-	*p* = 0.002
GeparDouze [52]	III	Atezolizumab + paclitaxel + carboplatin → doxorubicin/epirubicin + cyclophosphamide Adjuvant atezolizumab vs. Paclitaxel + carboplatin → doxorubicin/epirubicin + cyclophosphamide	775 vs. 775	4-year EFS	85.2% vs. 81.9%	0.80; 95% CI 0.62–1.03	*p* = 0.08
				4-year OS	90.2% vs. 89.5%	0.86; 95% CI 0.62–1.19	-
Create-X [53]	III	Adjuvant capecitabine group vs. observation group	455 vs. 455	5-year DFS	74.1% vs. 67.6%	0.70; 95% CI 0.53–0.92	*p* = 0.01
				5-year OS	89.2% vs. 83.6%	0.59; 95% CI 0.39–0.90	*p* = 0.01
SYSUCC-001 [54]	III	Low-dose maintenance capecitabine group vs. observation group	222 vs. 221	5-year DFS	82.8% vs. 73.0%	0.64; 95% CI 0.42–0.95	*p* = 0.03
				5-year DDFS	85.8% vs. 75.8%	0.60; 95% CI 0.38–0.92	*p* = 0.02
				5-year OS	85.5% vs. 81.3%	0.75; 95% CI 0.47–1.19	*p* = 0.22
GEICAM/2003-11_CIBOMA/2004-01 [54]	III	Extended adjuvant capecitabine group vs. observation group	448 vs. 428	5-year DFS	79.6% vs. 76.8%	0.82; 95% CI, 0.63–1.06	*p* = 0.136
				5-year OS	86.2% vs. 85.9%	0.92; 95% CI, 0.66–1.28	*p* = 0.623
OlympiA [55]	III	Adjuvant olaparib vs. placebo	921 vs. 915	3-year iDFS	85.9% vs. 77.1%	0.58; 99,5% CI 0.41–0.82	*p* < 0.001
				3-year DDFS	87.5% vs. 80.4%	0.57; 99,5% CI 0.39–0.83	*p* < 0.001
PARTNER [56]	II/III	Carboplatin + paclitaxel + olaparib → anthracycline-based chemotherapy vs. Carboplatin + paclitaxel → anthracycline-based chemotherapy	39 vs. 45	3-year EFS	96.4% vs. 80.1%	-	*p* = 0.04
				DDFS	96.4% vs. 87.9%	-	*p* = 0.20
				3-year OS	100% vs. 88.2%	-	*p* = 0.04

**Table 4:** Correlation Residual cancer burden (RCB) and Event-free survival (EFS) in Keynote-522 study.

RCB Category	Group	Events by Percentage of EFS	36-Month EFS	Risk Factor (95% CI)
RCB-0	Pembrolizumab	5.20%	94.70%	0.70 (0.38–1.31)
	Placebo	7.30%	92.60%	
RCB-1	Pembrolizumab	17.40%	84.40%	0.92 (0.39–2.20)
	Placebo	20.00%	83.80%	
RCB-2	Pembrolizumab	25.50%	75.70%	0.52 (0.32–0.82)
	Placebo	44.30%	55.90%	
RCB-3	Pembrolizumab	72.50%	26.20%	1.24 (0.69–2.23)
	Placebo	69.20%	34.60%	

## Data Availability

No new data were created or analyzed in this study.

## Supplementary Materials

The following supporting information can be downloaded at: https://www.mdpi.com/article/10.3390/cancers18111718/s1, Table S1: Examples of scales assessing response to systemic therapy in breast cancer after preoperative chemotherapy.
