# Supplementary material for: Pathological Complete Response After Neoadjuvant Chemotherapy in Breast Cancer: A Literature Overview
Source: Cancers (Basel). 2026 May 25;18(11):1718. doi: 10.3390/cancers18111718 (PMC13255888; doi:10.3390/cancers18111718)
Supplement: Supplementary file 1 [file cancers-18-01718-s001.zip › cancers-4285170-supplementary.pdf]

Table S1. Examples of scales assessing response to systemic therapy in breast cancer after preoperative chemotherapy

Assessment of response to systemic treatment in breast cancer		
Assessment according to Pinder		
	Breast:<br><br>1. pCR: (1) no residual cancer or (2) no residual invasive cancer but with carcinoma in situ<br><br>2. Partial response:<br><br>(1) minimal residual disease (< 10% residual cancer) or<br><br>(2) response with 10–50% residual cancer, or<br>(3) > 50% residual cancer with evidence of post-treatment damage<br><br>3. No evidence of response to treatment	Lymph nodes<br><br>1. No metastases and no evidence of response to treatment<br>2. No metastases, but evidence of response to treatment<br>3. Metastases present, but with evidence of response to treatment<br>4. Metastases present, but no evidence of response to treatment
Miller and Payne system	Grade 1: No change or abnormality in individual tumor cells, but no reduction in overall cellularity.<br>Grade 2: Minor tumor cell loss, but overall cellularity still high; loss of up to 30% of cells.<br>Grade 3: Estimated reduction of 30% to 90% in the number of tumor cells.<br>Grade 4: Significant tumor cell loss, such that only small clusters or widely scattered individual cells remain; loss of more than 90% of tumor cells.<br>Grade 5: No tumor cells in sections of the tumor site.<br>Ductal carcinoma in situ (DCIS) may be present.<br>Grade 1–4 is classified as partial pathologic response (pPR), and grade 5 as complete response. Pathological response<br>(cPR).	

Residual cancer burden (RCB)	Assessed tumor parameters:<br>Area of the primary tumor bed [mm] × [mm]<br>Total cancer cellularity (as a percentage of area)<br>(%)<br>Proportion of carcinoma in situ (%)<br>Lymph node assessment	Assessed nodal parameters<br><br>Number of positive lymph nodes<br>Diameter of the largest metastasis [mm]
